# A nationwide multi-institutional retrospective study to identify prognostic factors and develop a graded prognostic assessment system for patients with brain metastases from uterine corpus and cervical cancer

**DOI:** 10.1186/s12885-017-3358-6

**Published:** 2017-06-02

**Authors:** Nakamasa Hayashi, Hideaki Takahashi, Yuzo Hasegawa, Fumi Higuchi, Masamichi Takahashi, Keishi Makino, Masatoshi Takagaki, Jiro Akimoto, Takeshi Okuda, Yoshiko Okita, Koichi Mitsuya, Yasuyuki Hirashima, Yoshitaka Narita, Yoko Nakasu, On Behalf of the Committee of Brain Tumor Registry of Japan Supported by the Japan Neurosurgical Society

**Affiliations:** 1 Division of Neurosurgery, Shizuoka Cancer Center Hospital, Nagaizumi, Shizuoka, 411-8777 Japan; 2 Division of Gynecology, Shizuoka Cancer Center Hospital, Shizuoka, 411-8777 Japan; 3 Department of Neurosurgery, Niigata Cancer Center Hospital, Niigata, 951-8666 Japan; 4 Division of Neurological Surgery, Chiba Cancer Center, Chiba, 260-8717 Japan; 5 Department of Neurosurgery, Dokkyo Medical University, Tochigi, 321-0293 Japan; 6 Division of Neurosurgery, National Cancer Center, Tokyo, 104-0045 Japan; 7 Department of Neurosurgery, Kumamoto University, Kumamoto, 860-8555 Japan; 8 Department of Neurosurgery, Osaka Medical Center for Cancer and Cardiovascular disease, Osaka, 537-8511 Japan; 9 Department of Neurosurgery, Tokyo Medical University, Tokyo, 160-8402 Japan; 10 Department of Neurosurgery, Kinki University, Osaka, 589-8511 Japan; 11 Department of Neurosurgery, Osaka National Hospital, Osaka, 540-0006 Japan

**Keywords:** Brain metastasis, Graded prognostic assessment, Radiation, Surgery, Uterine cervical cancer, Uterine corpus cancer

## Abstract

**Background:**

The prevalence of brain metastases (BM) from uterine cancer has recently increased because of the improvement of overall survival (OS) of patients with uterine cancer due to its early detection and improved local control as a result of new effective treatments. However, little information is available regarding their clinical characteristics and prognosis, because oncologists have encountered BM from uterine cancer on rare occasions.

**Methods:**

Records from 81 patients with uterine BM were collected from 10 institutes in Japan. These were used in a multi-institutional study to identify prognostic factors and develop a graded prognostic assessment (GPA) for patients with BM from uterine cancer.

**Results:**

Median OS after the development of BM was 7 months (95% confidence interval, 4 to 10 months). Multivariate analysis revealed that there were survival differences according to the existence of extracranial metastases and number of BM. In the present uterine-GPA, a score of 0 was assigned to those patients with ≥5 BM and extracranial metastasis, a score of 2 was assigned to those patients with one to four BM or without extracranial metastasis, and a score of 4 was assigned to those patients with one to four BM and without extracranial metastasis. The median OS for patients with a uterine-GPA scores of 0, 2, and 4 was 3, 7, and 22 months, respectively. A survival analysis confirmed the presence of statistically significant differences between these groups (*p* < 0.05). The results were validated by data obtained from the National Report of Brain Tumor Registry of Japan.

**Conclusion:**

Uterine GPA incorporates two simple clinical parameters of high prognostic significance and can be used to predict the expected survival times in patients with BM from uterine cancer. Its use may help in determining an appropriate treatment for individual patients with BM.

## Background

The prevalence of brain metastases (BM) from uterine cancer has increased because of the improvement of overall survival (OS) of patients with uterine cancer due to its early detection and improved local control as a result of new effective treatments [1–7]. However, because of the rarity of BM from uterine cancer, little is known regarding its clinical characteristics, optimal management, and prognosis.

BM are usually treated with multimodal therapy using a combination of whole brain radiotherapy (WBRT), stereotactic radiosurgery (SRS), and surgical resection. Although BM from uterine cancer was reportedly associated with poor prognosis, with a median survival ranging from 1 to 8 months, some authors strongly suggested that surgery was an effective treatment for solitary BM in patients with uterine cancer and that postoperative radiation therapy also prolonged survival [1–3, 5, 6, 8–12]. Recently, Chung reported that SRS could be an efficient palliative measure to relieve neurological symptoms caused by BM from uterine cancer. The median survival time in the patient group undergoing SRS and WBRT was significantly longer than that in the patient group undergoing SRS alone [1]. Clarification of the clinical characteristics of patients who would benefit from surgery and/or radiation is an important and urgent matter. An optimal therapeutic guideline or prognostic scale should be established to enable an estimation of survival times and the selection of appropriate treatments for patients with BM from uterine cancer.

The prognostic factors for patients with BM vary according to the primary diagnosis, and a diagnosis-specific graded prognostic assessment (GPA) has been developed for use in several primary metastatic tumors [13–15]. GPA has not yet been developed for BM from uterine cancer. Here we performed a nationwide multi-institutional study to evaluate the prognostic factors of BM from uterine cancer and have developed a diagnosis-specific GPA. This was validated by data obtained from the Report of Brain Tumor Registry of Japan.

## Methods

The present study was a multi-institutional retrospective analysis of 81 patients with BM from uterine cancer from 10 institutions in Japan between April 2002 and March 2014. Approval for this study was obtained from the institutional research ethics board of Shizuoka Cancer Center (T27-23-27-1-5). Data obtained from the Report of Brain Tumor Registry of Japan, including 2907 patients with BM who newly started treatment from 2001 to 2004, was used as a validation set [16]. Individual written informed consent was waived because this study was retrospective in design and based on database extracted records.

The clinical data obtained included the date of birth, primary cancer site, histological type, date of the original cancer diagnosis and presence of BM, whether the primary lesion was controlled at BM diagnosis, date and type of the initial treatment for BM, date and type of salvage therapy (if any) for BM, date of death or last follow-up visit, Karnofsky performance status (KPS) at initial diagnosis of BM, number and maximum size of BM, and whether extracranial metastases were present.

OS was calculated from the date of diagnosis of BM to death of any reason or the last day of follow-up according to Kaplan-Meier estimates. Prognostic factors were analyzed using the log-rank test for univariate analysis and Cox regression analysis for multivariate analysis. A *p* value <0.05 was considered to indicate a statistically significant difference. Only statistically significant prognostic factors were used in the determination of GPA. Analyses were performed using the JMP® software (Version 11, SAS institute Inc., Tokyo, Japan).

## Results

### Patient characteristics

A total 81 patients were enrolled, and their characteristics are listed in Table 1. The primary origin of the tumor was the uterine corpus in 48 patients (59%) and the uterine cervix in 33 patients (41%). The median age at diagnosis of BM was 59 years. The most common tumor histology was adenocarcinoma in 71% of the patients with uterine corpus cancers, and squamous cell carcinoma in 58% of those with uterine cervical cancers. The primary tumor was controlled in half of the patients. Fifty-nine patients (73%) had extracranial metastases with the lung being the most frequently involved organ (*n* = 43) followed by the lymph nodes (*n* = 36), bone (*n* = 15), and liver (*n* = 10). The median time from diagnosis of the primary uterine cancer to the appearance of BM was 25 months. BM were detected in 4 patients (5%) prior to the diagnosis of uterine cancer. Twenty-eight, 30, 12, and 7 patients had a solitary, 2–4 lesions, 5–9 and ≥10 lesions, respectively. Four patients with uterine cervical cancer suffered from meningeal carcinomatosis. The site of BM was only supratentorial in 45 patients. Infratentorial involvements were found in 32 patients. KPS was <70% in 38 (47%) patients.

**Table 1:** Clinical characteristics of patients with brain metastasis of uterine cancer

	Study cohort				Validation cohort	
	No. (%)	Uterine corpus cancer	Uterine cervical cancer	*p*-value	No. (%)	*p*-value
	81	48	33		43	
Median age (range)	59 years (26-84)	60.5 (26-84)	58 (33-80)		56 (29-80)	
< 65	51 (63)	27 (56)	24 (73)	0.13	34 (79)	0.06
≥ 65	30 (37)	21 (44)	9 (27)		9 (21)	
Histology						
Adenocarcinoma		34	8			
Squamous cell carcinoma			19			
Carcinosarcoma		5				
Small cell carcinoma			3			
Others		7	2			
NA		2	1			
Primary tumor status				0.79		
Controlled	38 (47)	22 (46)	16 (48)			
Uncontrolled	39 (48)	23 (48)	16 (48)			
NA	4	3	1			
Extracranial metastasis				0.35		0.05
Yes	59 (73)	36 (75)	23 (70)		25 (58)	
No	17 (21)	8 (17)	9 (27)		9 (21)	
NA	5	4	1		9	
Mean time to BM	25 months (−11-130)	25 (−5-130)	33 (−11-114)	>0.05	28 (−6-126)	>0.05
Number of BM				0.35		
1	28 (35)	20 (42)	8 (24)		26 (60)	**0.01**
2-4	30 (37)	15 (31)	15 (45)		14 (32)	
5-9	12 (15)	8 (17)	4 (12)		2 (5)	
≥ 10	7 (9)	5 (10)	2 (6)		1 (2)	
meningeal carcinomatosis	4 (5)	0	4 (12)		0	
Site of BM				0.16		0.19
supratentorial only	45 (56)	31 (65)	14 (42)		30 (70)	
infratentorial involvement	32 (40)	17 (35)	15 (45)		13 (30)	
Karnofsky performance status				0.66		0.2
90-100%	9 (11)	3 (6)	6 (18)		10 (23)	
70-80%	29 (36)	18 (38)	11 (33)		17 (40)	
< 70%	38 (47)	24 (50)	14 (42)		13 (30)	
NA	5	3	2		3	
Recursive Partitioning Analysis				**0.02**		
Class I	4 (5)	0	4 (12)			
Class II	34 (42)	21 (44)	13 (39)			
Class III	38 (47)	24 (50)	14 (42)			
NA	5	3	2			

*BM* brain metastases

*P*-value are calculated using the chi-square test

Significant values are in bold font

According to the Recursive Partitioning Analysis (RPA), only four patients (5%) with uterine cervical cancer were categorized as class I whereas 38 patients (47%) were categorized as class III. There were no statistical differences concerning the patient baseline characteristics, with the exception of the RPA class between those patients with primary uterine corpus cancer and those with primary uterine cervical cancer.

**Fig. 1:**
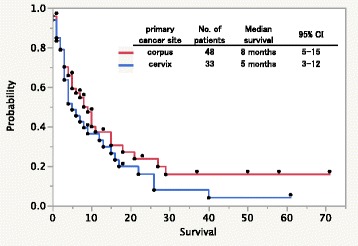
Kaplan-Meier survival curves in patients with brain metastases from uterine cancer

The median OS of all patients was 7 months [95% confidence interval (CI) 4–10 months]. The median OS was 8 months [95% CI 5–15 months] for uterine corpus cancer, and 5 months [95% CI 3–12 months] for uterine cervical cancer. Kaplan-Meier survival curve for primary site and survival months are presented in Fig. 1; log-rank test for the primary site and survival was not significant (*p* = 0.239).

### Treatment

Thirty patients (37%) underwent surgical excision of their BM where the maximum diameter of the tumor was ≥24 mm. Twenty-eight of these patients (93%) underwent WBRT after surgery, and only two patients underwent surgery alone. Radiation therapy was the main treatment in 45 patients. This included WBRT (*n* = 24), local radiation (*n* = 1), and stereotactic radiotherapy (*n* = 23). Four of these patients received an Ommaya reservoir, whereas one patient underwent ventriculoperitoneal shunt surgery. Twenty-three of the 31 patients with <5 BM were treated by stereotactic radiotherapy, whereas all 14 patients with ≥5 BM were treated using WBRT. Three of the four patients with meningeal carcinomatosis were treated by WBRT combined with intrathecal chemotherapy, and only one patient was treated by intrathecal chemotherapy alone. Two patients underwent supportive treatment only.

### Prognostic factor analysis

KPS at initial diagnosis of BM, number of BM, and existence of extracranial metastases were significant prognostic factors for OS in univariate analysis. The median OS was significantly prolonged in those patients who underwent surgical excision and irradiation compared with that of patients who underwent only radiation, surgery, or chemotherapy or who were just observed.

Multivariate analysis was performed incorporating the factors that were significant in the univariate analysis. The results showed that there were survival differences according to the existence of extracranial metastases, number of BM, and treatment received by the patient (Table 2).

**Table 2:** Multivariate Cox regression model for overall survival

	Hazard ratio	95%CI	p
Existence of extracranial metastases			
yes vs no	2.667	1.32-5.91	**0.0052**
KPS at initial diagnosis of BM			
< 70 vs ≥70	1.487	0.86-2.58	0.1525
Number of BM			
2-4 vs 1	1.009	0.49-2.06	0.9801
≥ 5 vs 1	2.440	1.16-5.16	**0.0184**
≥ 5 vs 2-4	2.418	1.20-4.94	**0.0135**
Treatment			
Surgery only vs Surgery + Radiation	15.84	2.24-70.51	**0.0100**
Surgery only vs Radiation	11.19	1.60-48.16	**0.0199**
Radiation vs Surgery + Radiation	1.416	0.79-2.62	0.2436

Significant values are in bold font

### Uterine-GPA

**Fig. 2:**
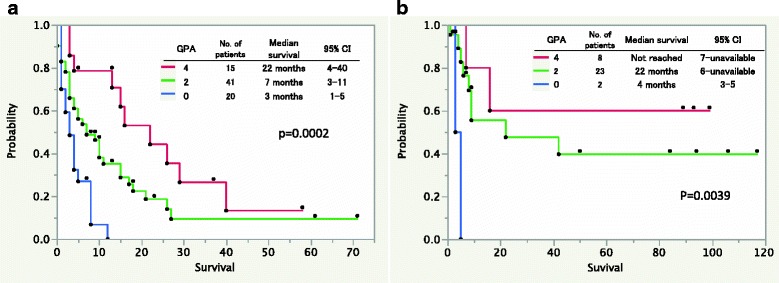
**a**: Kaplan-Meier survival curves in the study cohort according to the new graded prognostic assessment for patients with uterine cancer. **b**: Kaplan-Meier survival curves in the validation cohort according to the graded prognostic assessment for patients with uterine cancer

Table 3 summarizes the GPA indices for the patients. The GPA for uterine cancer uses two prognostic factors. A score of 0 was assigned to those patients with ≥5 BM and extracranial metastasis, a score of 2 was assigned to those patients with one to four BM or without extracranial metastasis, and a score of 4 was assigned to those patients with one to four BM and without extracranial metastasis. Because the hazard ratio of the numbers of BM and existence of extracranial metastasis were equivalent, the weight of the assigned score was equal among these factors. The median OS for patients with a uterine-GPA scores of 0, 2, and 4 was 3, 7, and 22 months, respectively. A survival analysis confirmed the presence of statistically significant differences between these groups (*p* < 0.05, Fig. 2a).

**Table 3:** Definition of graded prognostic assessment for patients with brain metastasis from uterine cancer

Significant prognostic factors	GPA scoring criteria	
	2	0
Number of BM	1-4	≥5
Extracranial metastasis	No	Yes

### Validation of the uterine-GPA

The validation dataset obtained from the Report of Brain Tumor Registry Japan consisted of results from 43 patients with BM from uterine cancer (Table 1) [16]. The number of BM in these patients was significantly lesser than that found in the current study cohort. The uterine-GPA could be assessed in 33 of the 43 patients. A score of 4 (eight patients, median OS, not reached; 95% CI, 7-unavailable) correlated with a good prognosis, a score of 2 (23 patients, median OS, 22 months; 95% CI, 6-unavailable) correlated with an intermediate prognosis, and a score of 0 (two patients, median OS, 4 months; 95%CI, 3–5) correlated with a poor prognosis. The differences between the groups were statistically significant (*p* < 0.05, Fig. 2b).

## Discussion

The present study was performed to evaluate the prognostic factors of BM from uterine cancer using the case registration method from 10 Japanese institutions. We have developed the first diagnosis-specific GPA for uterine cancer, based on the independent prognostic factors. According to the original GPA, a score of 4 correlated with the best prognosis, and a score of 0 correlated with the worst prognosis [15]. This uterine-GPA enabled the prediction of the expected OS times in patients with BM from uterine cancer. Its use may help future clinical decisions in determining the appropriate treatment for individual patients with BM.

Review articles reported that BM from uterine cervix and corpus cancers were rare, with only 115 patients documented in 35 papers and 96 patients in 34 papers before 2012, respectively [5, 6]. The most frequent sites of distant metastasis were the lung, bone, and liver. These papers included reports on individual cases or relatively small numbers of patients (2–20 patients), and there were no reports on large numbers of patients. Recently, a Korean study provided a clinical analysis of BM in gynecologic cancers, including 29 patients with uterine cancer [4]. Although the number of patients per institution was not large, (1 to 30 over 10 years), the current study of 81 patients is the largest investigation of the occurrence of BM in patients with uterine cancer.

BM are considered to be part of a disseminated disease process and their occurrence is a late event in the course of the disease [5, 9]. The prevalence of BM has, therefore, increased because of the prolonged survival of patients [3]. Chura reported that the majority of the patients (16 of 20 patients, 80%) also had evidence of other metastatic disease at the time of diagnosis of BM [2].

BM from uterine cancer is associated with a poor prognosis with limited survival in spite of the use of modern multimodal treatment options. Here the median survival after the diagnosis of BM was 7 months and was comparable to previous reports describing median survivals ranging from 1 to 8 months [1–3, 5, 6, 8–12]. Several clinical characteristics influencing the survival of patients have been reported. Kim recently reported improved survival times of 23.3 months for uterine corpus cancer and 8.8 months for uterine cervical cancer [4]. They stressed that the presence of solitary BM (44.5%), small BM (<2 cm; 21.2%), absence of pulmonary (56.2%) and extracranial (24.1%) disease as well as good performance status were associated with good prognosis. Mahmoud-Ahmed reported that the patients with multiple BM had a shorter survival than those with a single metastasis [12]. Chura reported that the median survival times for patients with isolated BM and no systemic disease was better than that for those with systemic disease [2]. Recently, Divine also reported that isolated BM from gynecologic malignancies was significantly associated with survival [17].

Some authors strongly suggested that surgery was an effective treatment for solitary BM in patients with uterine cancer and that postoperative radiation therapy also prolonged survival [9, 11, 12]. Recently, Kimyon reported that surgical resection with radiation improved the survival for isolated BM from endometrial cancer [18]. Recent retrospective study of patients with gynecological malignancies showed that treatment with multimodal therapy including surgical resection and radiation might prolong overall survival [19]. The present study also revealed the advantage of surgery followed by radiation therapy.

The potential use of a GPA is to select patients with good prognosis in order to give aggressive treatments to the patients who would most benefit from. The present study revealed that the absence of extracranial metastases, and small numbers of BM were independent factors for improved OS in patients with BM from uterine cancer. A patient with 1–4 BM from uterine cancer and without extracranial metastasis, i.e. with a GPA score of 4, would most benefit from aggressive treatments.

The present study has limitations that are inherent in a retrospective design and the use of a small patient cohort with a rare type of BM.

## Conclusions

The proposed uterine-GPA incorporates two simple clinical parameters, the existence of extracranial metastases and the number of BM that are of high prognostic significance. This information enables the prediction of the expected survival times in patients with BM from uterine cancers. It may help in deciding the appropriate intensity and timing of treatment for individual patients with BM.

## Abbreviations

BM: Brain metastases
CI: Confidence interval
GPA: Graded prognostic assessment
KPS: Karnofsky performance status
OS: Overall survival
RPA: Recursive partitioning analysis
SRS: Stereotactic radiosurgery
WBRT: Whole brain radiotherapy
